# A Review of Plant Selenium-Enriched Proteins/Peptides: Extraction, Detection, Bioavailability, and Effects of Processing

**DOI:** 10.3390/molecules28031223

**Published:** 2023-01-26

**Authors:** Yangyang Xiong, Yatao Huang, Lin Li, Yanfang Liu, Liya Liu, Lili Wang, Litao Tong, Fengzhong Wang, Bei Fan

**Affiliations:** 1 Institute of Food Science and Technology, Chinese Academy of Agricultural Sciences, Beijing 100193, China; 2 Key Laboratory of Agro-Products Quality and Safety Control in Storage and Transport Process, Ministry of Agriculture and Rural Affairs, Beijing 100193, China

**Keywords:** Se-enriched proteins/peptides, extraction, bioavailability, processing, bioactivity

## Abstract

As an essential trace element in the human body, selenium (Se) has various physiological activities, such as antioxidant and anticancer activity. Selenium-enriched proteins/peptides (SePs/SePPs) are the primary forms of Se in plants and animals, and they are the vital carriers of its physiological activities. On the basis of current research, this review systematically describes the extraction methods (aqueous, alkaline, enzymatic, auxiliary, etc.) and detection methods (HPLC–MS/MS, GC–ICP-MS, etc.) for SePs/SePPs in plants. Their bioavailability and bioactivity, and the effect of processing are also included. Our review provides a comprehensive understanding and theoretical guidance for the utilization of selenium-enriched proteins/peptides.

## 1. Introduction

Selenium (Se) is a nonmetallic element that exists in nature and can be broadly divided into two categories: inorganic (selenite, selenate, etc.) and organic (selenoprotein, selenopeptide, selenoamino acid, selenopolysaccharide, etc.). The latter is formed by the biological transformation of Se, among which selenium-enriched proteins/peptides (SePs/SePPs) account for the majority. Se deficiency can cause Creutzfeldt–Jakob disease, macrosomia, and many other diseases; thus, moderate intake of Se is beneficial [1]. In the human body, Se is used to synthesize selenoproteins, which have diverse functions. Selenoproteins, which have antioxidant activity, play a vital role in maintaining redox homeostasis; they also participate in signal transduction, cell proliferation, transformation, immunity, and many other biological processes [2]. The nutritional health effects of Se on the human body mainly depend on its form [3]. Highly nucleophilic Se amino acids, particularly its peptide sequence, and good bioavailability are the main reasons for the good biological activity of SePs/SePPs [4].

SePs/SePPs are mainly extracted from organisms. Compared with animals and microorganisms, they are easier to acquire and extract from plants. Therefore, it is essential to research the extraction and preparation, detection and identification, digestion and absorption, exploitation and utilization, and other aspects of SePs/SePPs in plants. Liquid-phase and enzymatic extraction are commonly used methods of SeP/SePP extraction. The extraction efficiency can be improved by heat, ultrasound, microwave, pressurization, and other auxiliary treatments. There are various forms of SePs/SePPs, and the different ways that Se binds to proteins and peptides affect their spatial structure, bioavailability, bioactivity, and other properties. Liquid chromatography (LC), gas chromatography (GC), and capillary electrophoresis (CE) can be used for the separation of SePs/SePPs. In combination with inductively coupled plasma mass spectrometry (ICP/MS), electrospray ionization mass spectrometry (EI/MS), and atomic fluorescence spectrometry (AFS), the forms of Se can be analyzed and identified.

Bioavailability is a vital indicator of whether SePs/SePPs can function effectively in living organisms [5]. The processing method will affect the content and structure of SePs/SePPs in plants, and then affect their bioavailability; especially after processing, their loss can be profound. In addition, there are various functional activities of SePs/SePPs with different forms of Se or binding states. The nature and sequence of amino acids, the differences between the C- and N-terminals, the length of the peptide chain, and hydrophobicity can all affect the biological activities of SePs/SePPs. However, there is a lack of systematic studies in this area [4]. The bioaccessibility of Se varies greatly depending on the form [6]. On these aspects, the processes of extracting and preparing SePs/SePPs are significant for the healthy function of Se.

In this paper, we sort out the relevant reports in recent years, including those on the extraction, detection, digestion, and utilization of plant SePs/SePPs, the effect of processing, and their bioavailability and bioactivity, with the purpose of inspiring further research on their use on a larger scale and promoting further development.

## 2. Preparation and Detection of Se-Enriched Proteins/Peptides

### 2.1. Extraction and Preparation of Plant Se-Enriched Proteins/Peptides

#### 2.1.1. Liquid-Phase Extraction

The extraction of SePs is not only indispensable for further research and utilization, but also a prerequisite for the preparation of SePPs. Liquid-phase extraction methods are often used; common ones include water extraction, alkali extraction, salt extraction, alcohol extraction, and buffer solution extraction, all of which have advantages and disadvantages (Table 1). Thus, the most suitable method needs to be selected on the basis of practical application.

#### 2.1.2. Enzymatic Extraction

Because of the mild conditions, favorable specificity, and excellent activity, enzymatic extraction is widely used in protein extraction and peptide preparation. Commonly used enzymes include trypsin, pepsin, proteinase K, and proteinase XIV. However, the extraction of proteins by enzymes alone is time-consuming. In practice, protein extraction is usually carried out using a combination of enzymes, and it is often combined with other methods to enhance the extraction rate.

Enzymes are essential to break down the obtained proteins into lower-molecular-weight peptides. Fang et al. [18] used alkaline protease, neutral protease, trypsin, and pepsin to hydrolyze the SePs of rice, and SePPs with different degrees of hydrolysis were obtained. Zhu et al. [19] used alkaline protease and neutral protease to break down *Cardamom violifolia* SePs into SePPs. Zhang et al. [20] used alkaline protease, neutral protease, and papain in a ratio of 2:1:1 to prepare soy SePPs, and the degree of hydrolysis was 68.53% under optimal conditions. The specific nature of enzymes and the structure of the cell walls of the plant hinder the solubilization of enzymatic products [11]. In most cases, researchers do not use the enzymatic method to prepare SePs directly, but use alkaline for preparation and enzymes to disintegrate the SePs into SePPs.

#### 2.1.3. Auxiliary Extraction

Compared with liquid-phase or enzymatic extraction, auxiliary extraction can promote the extraction efficiency of proteins/peptides. Frequently used methods of auxiliary extraction include heating, stirring, microwave, ultrasound, and high pressure. Regardless of the extraction solution used, ultrasound improved the efficiency of extracting SePs from *Allium sativum* and *Brassica juncea*, and maintained good Se recovery (75–120% of the total Se in the plant) [21]. Compared with conventional extraction, ultrasound has preferable effectiveness in extracting *Cardamine violifolia* SePs, with 77% purity of protein powder and 9097.33 ± 35.66 mg/kg of total Se [22]. Microwaves can significantly promote the enzymolysis of SePs and produce SePPs [23]. Guzmán et al. [24] combined microwave with enzymatic extraction; after optimizing the related conditions, the optimal extraction recovery of total Se was 80% ± 4%. Combining ultrahigh pressure (300 MPa, 5 min) with alkali, the total Se content of the SePs of obtained peanut powder was 0.37 mg/kg, including 0.35 mg/kg of organic Se, which was about 95% of the total [25]. In the preparation of tea SePs, the alkali was combined with freezing–thawing to obtain crude SePs [26]. Freezing–thawing allows cell-wall fragmentation, making SePs more readily available. The final extraction rate of the crude SePs was 60.93%, and the purity was 52.07%.

### 2.2. Separation and Detection of Se-Enriched Proteins/Peptides

The form of Se is closely related to its bioavailability and bioactivity. Organic Se is better than inorganic Se in terms of safety and bioavailability. Most organic Se exists in proteins and peptides in the form of seleno-substituted amino acids (Table 2) [5]. Therefore, detecting the form of SePs/SePPs is essential for functional research or product development. There are several methods for separating and detecting SePs/SePPs, which have different sensitivity and applicability, and their selection needs to be adjusted according to the actual situation.

#### 2.2.1. Separation Methods

##### Liquid Chromatography

As a separation technique, liquid chromatography (LC) uses a liquid as the mobile phase to elute the sample, while the stationary phase can take many forms. LC is widely used in the detection of Se forms and is often combined with other detection techniques, including high-performance liquid chromatography (HPLC), reversed-phase ion pair HPLC (RP-IP-HPLC), ion exchange chromatography (IEC), and size-exclusion chromatography (SEC), which are mainly used for the separation of SePs. 

Among them, HPLC has shown good adaptability for SeP separation [27], and it can be used to separate SePs from various types of plants, such as green tea [28] and radish sprouts [29]. Cubadda et al. [30] used RP-IP-HPLC to separate wheat SePs after ultrasound-assisted enzymatic extraction, with final methionine Se and selenate accounting for 72–85% and 2–6% of the total Se content, respectively. In the separation of rice SePs, RP-IP-HPLC was more sensitive and performed better than anion-exchange chromatography when 0.5 mM tetrabutylammonium hydroxide and 10 mM ammonium acetate (pH 5.5) were used as mobile phases [31]. Separation of SeCys, SeMet, Se(IV), and Se(VI) using IEC was also performed [32]. Ayouni et al. [33] completed the separation of organic and inorganic Se, Csapó et al. [34] completed the separation of SeCys from SeMet, and Zhang et al. [35] successfully isolated SePs from plant aqueous extracts. The results of Moreno et al. [36] showed that the effective separation range of IEC was 7–0.1 kDa, while the effective separation range of SEC was 300–1 kDa, indicating that size-exclusion chromatography is suitable for separating SePs with relatively large molecular weights. Similarly, Kapolna et al. [37] separated SePs with two molecular weights in chives using SEC and RP-IP-HPLC. Coupling SEC with HPLC, Tie et al. [17] used SEC to concentrate crude SePs extract of *Flammulina velutipe* to reduce interference with the target components, and then used HPLC for further separation.

##### Gas Chromatography

Gas chromatography (GC) is a method that uses gases as mobile phases, mainly for analyzing various gases and separating volatile organic substances. GC is suitable for the separation of dimethyl selenium (DMSE), diethyl selenium (DESE), dimethyl diselenium (DMDSe), and hydrogenated dimethyl sulfide (d6-DMS), which are volatile organic Se molecules in plants [38], while the derivatization method is required for the separation of Se amino acids and other substances. For example, Lee et al. [39] used alkyl chloroformate derivatization to analyze the Se-free amino-acid composition of Se-enriched broccoli. GC can be used for ultra-trace-level detection of Se, but it requires special detection conditions such as reagents, sample processing, and interference sources [40]. Nakashima et al. [41] used GC to detect Se content, and the limit of detection was 0.04 μg. Due to the relatively cumbersome operation of the derivatization method, GC is used less frequently in determining nonvolatile organic selenium.

##### Capillary Electrophoresis

Capillary electrophoresis (CE) is a method that uses a high-voltage electric field as the driving force and a capillary tube as the separation channel to achieve separation. It can separate samples according to the differences in the shunt and distribution behavior of each group. 

CE also has an enormous scope of application in the separation of Se compounds. CE separation is based on the difference between the electrophoretic mobility and electroosmotic transport of target analytes, and the two transport phenomena can be in the same or opposite direction depending on the experimental conditions [42]. In one experiment, when the injection argon pressure was 0.04 MPa, the injection time was 10 s, and the sample volume was about 0.3 mL for a capillary tube with inner and outer diameters of 75 and 340 mm, ginger SeMet, SeCys_2_, Se(IV), and Se(VI) were effectively separated [43]. Similarly, Zhao et al. [44] separated Se(VI), Se(IV), SeCys_2_, and SeMet in rice using the reverse CE mode. CE is often used as an alternative or complementary technique to HPLC. However, the relatively small injection volume and the high sensitivity requirement for the backend detector limit its application in Se morphology analysis to some extent [45].

#### 2.2.2. Testing and Identification Methods

##### Mass Spectrometry

Mass spectrometry (MS) uses electric and magnetic fields to separate moving ions according to their mass-to-charge ratio and then detects them. Inductively coupled plasma MS (ICP-MS) and electrospray ionization MS (ESI-MS) are commonly used to detect Se forms. Because of the destructive nature of ICP, only known forms of Se can be detected. On the other hand, ESI is gentler; thus, it is possible to analyze unknown Se forms by molecular weight and fragment, which has many applications in SeP/SePP identification.

ICP-MS can be used in combination with a variety of separation techniques, such as HPLC, GC, CE, and SEC. Sugihara et al. [46] used HPLC–ICP-MS to detect Se forms in Se-enriched sprouts, and the main species found was SeMeSeCys. Thosaikham et al. [47] combined ICP-MS with RP-IP-HPLC to establish a method for determining Se forms in Se-enriched pak choi, with the limit of detection (LOD) and limit of quantification (LOQ) for each form (Se(VI), Se(IV), SeCys_2_, SeCys, SeMeSeCys, and SeMet) being less than 5 and 16 ng Se/mL, respectively. Zhao et al. [44] developed a CE-ICP-MS method for determining trace Se(VI), Se(IV), SeCys_2_, and SeMet in rice within 18 min, with detection limits of 0.1–0.9 ng Se/mL and recovery of 90–103%.

ESI-MS processes the sample by soft ionization, allowing the molecular weight and molecular formula of compounds to be determined in order to realize qualitative and quantitative analysis of SePs/SePPs. It offers higher sensitivity and resolution than ICP-MS and does not fragment sample molecules. Zagrodzki et al. [48] used HPLC–ESI-MS to analyze Se forms in kale. After separation by hydrophilic interaction liquid chromatography, the same forms of Se detected by HPLC–ICP-MS were found by ESI-MS under the same chromatographic conditions. The structure of the species was elucidated by molecular ion fragmentation, and previously unreported low-molecular-weight Se metabolites and derivatives were detected. ESI-MS is capable of quantifying and characterizing SeP/SePP forms in many plants, such as soybeans [49], cereals [50], carrots [51], and sprouts [29].

##### Hydride Generation Atomic Fluorescence Spectrometry

Using hydride generation atomic fluorescence spectrometry (HG-AFS), the hydride of the element being tested is thermally decomposed while the measured element dissociates into ground-state atomic vapor, which can be detected. It has less interference and is easy to operate [52], and it can also be coupled with HPLC. HPLC–HG-AFS is commonly used to analyze SeMet, SeCys, Se(IV), and Se(VI), and it shows high sensitivity when generating gaseous Se hydride H_2_Se [53]. Erzsébet et al. [54] used this method to detect SeMet and total Se content in Brazil nuts, which were quantified as 79.9 µg·g^−1^ (calculated as Se) and 82.9 µg·g^−1^, respectively. Ipolyi et al. [55] found that the detection limit of Se(IV) is 1.0 pg. The HPLC–HG-AFS system is not applicable for direct determination of Se(VI), although it provides a good detection limit compared to some detection methods. Grijalba et al. [56] coupled HG-AFS with RP-HPLC and used different ionic liquids as mobile-phase improvers for the separation and determination of Se(IV), Se(VI), SeMet, and SeMeSeCys, achieving complete separation in 12 min; this process has been successfully applied in the morphological analysis of Se in complex samples such as wine, beer, yeast, and garlic.

#### 2.2.3. Other Methods

Electrochemical detection (ED) applies a constant potential voltage between two electrodes. When the electroactive component passes over the electrode surface, a redox reaction occurs and the potential changes, thus achieving separation and detection of each analyte. It is commonly used for metal elements and transition elements. ED includes electrochemical titration, ion-selective electrode, voltammetry, and polarimetry. Voltammetry is the most widely used method because of its sensitivity and speed [57]. The results vary depending on the electrolyte used. For Se(IV), sulfuric acid performed better than perchloric acid and potassium chloride media when platinum and gold electrodes were used with a pre-enrichment time of 60 s, and the Se concentration varied linearly in the range of 5–15 μM and 0.1–10 μM, with a detection limit of 25 nM [58], and it could be developed for trace analysis. Cavalli et al. [59] combined anion-exchange chromatography with ED to analyze Se amino acids and similarly concluded that SeCys and SeMet were better detected under acidic medium conditions. Recovery was above 90% for both. Meanwhile, Se amino acids could be separated under alkaline conditions, but the baseline tended to drift. Since only the oxidized state of Se(IV) is electroactive, most electrochemical detection assays are centered on it; other forms of Se, while also detectable, have relatively fewer applications [57].

## 3. Bioaccessibility and Bioavailability of Se-Enriched Proteins/Peptides

**Figure 1:**
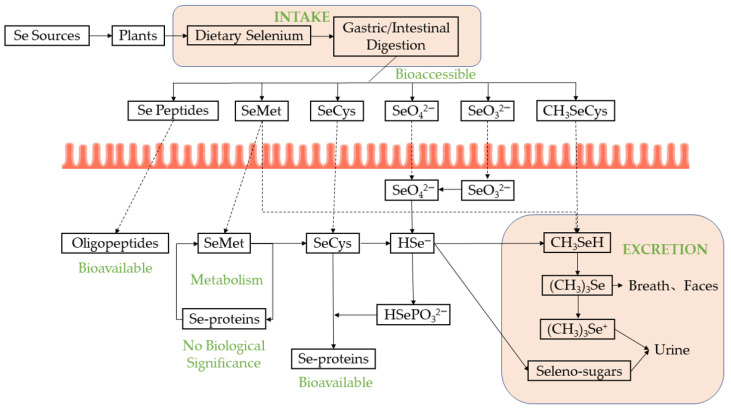
Absorption and metabolic pathways of selenium in humans [2,4,5].

There are several processes that SePs/SePPs need to go through, including intake, absorption, and metabolism (Figure 1). Absorption and utilization by the body are critical for physiological functions. Accordingly, it is essential to evaluate bioaccessibility and bioavailability. Bioaccessibility refers to the number of bioactive molecules released from the food matrix through the gastrointestinal tract that can eventually be absorbed by the small intestine for subsequent bioprocessing. It can be assessed by in vitro methods, and the quantity of compounds after digestion is used to estimate the effectiveness of intestinal absorption. Bioavailability refers to the number of compounds that can be further absorbed through the body’s mucosa for later use in normal physiological functions or storage in the circulatory system after metabolic processing and digestive release in the intestine. It includes gastrointestinal digestion, absorption and metabolism, tissue distribution, and bioactivity, and it can be assessed by in vivo analysis of metabolites in blood or urine [60,61].

Bioaccessibility represents potentially available components, and bioavailability reflects differences in bioaccessibility [62]. Simulated human gastrointestinal digestion experiments, in vitro cell culture experiments, and mouse experiments are often used to evaluate bioaccessibility and bioavailability. In vitro experiments, especially those using simulated human gastrointestinal digestion, are more focused on bioaccessibility (Table 3). To further investigate the digestion process of SePs/SePPs, they should be combined with in vivo experiments for verification.

Se enters the body by dietary, after digestion through the gastrointestinal tract. Seleno-substituted amino acids such as SeMet and SeCys follow the amino acid uptake mechanism, and are used for subsequent selenoprotein synthesis; SeO_3_^2−^ is absorbed by simple diffusion, while SeO_4_^2−^ is absorbed by the body through Se/OH^−^ cotransport. All the above components are converted to H_2_Se in the liver and can be excreted in various forms through respiration and urine.

### 3.1. In Vitro Evaluation

In vitro simulations of human gastrointestinal digestion often imitate the organs of the oral cavity, stomach, and small intestine. They can be divided into static and semi-dynamic. In static simulations, the experimenter is responsible for manipulating the entire process, mainly managing enzymes and controlling reaction time. Semi-dynamic gastric simulation is mechanistic, including mastication and the automatic addition of enzymes and digestive juices [73]. Ari et al. [74] studied Se in hydroponically grown leeks. The results showed that Se in leeks cultured with selenite had better bioavailability but lower bioaccessibility than those cultured with selenate. Because the inorganic Se in leeks cultivated with selenite only accounted for about 8%, MeSeCys and SeMet accounted for about 60% and 11%, respectively. Oliveira et al. [65] used a similar approach to study the SePs of red bean sprouts, and they found that Se was released in vitro in the gastric phase due to the proteolytic action of pepsin. Bile and trypsin (a mixture of protease, amylase, and lipase) promote secondary peptide hydrolysis, while Se does not significantly affect the bioavailability of other elements.

Cellular experiments are often mediated by Caco-2 cells, which are structurally and functionally similar to differentiated small intestine epithelial cells. They have structures such as microvilli and contain enzymes associated with the epithelial brush border of the small intestine. They can be used to perform experiments that imitate intestinal transit in vivo and assess bioavailability. Delaqua et al. [68] studied the digestion of Se in wheat and showed that only 19.6% of the Se content, on average, was absorbed by the human intestinal epithelium, while lower concentrations were better absorbed. Similar results were shown in the study of Zhang et al. [70], with values for Se bioavailability of 0.65% ± 0.13% and 0.52% ± 0.33% for selenite at 4 and 20 μM, respectively (*p* > 0.05), and 4.01% ± 1.145% for SeMet at low doses and 8.01% ± 1.63% at high doses, which is significantly higher (*p* < 0.05). The highest bioavailability of SePPs was found in Caco-2 cells at 9.476% ± 0.97%, with no significant difference between low and high doses (*p* > 0.05).

### 3.2. In Vivo Evaluation

In vivo evaluation is mainly performed using mice in simulated experiments. Yan et al. [72] evaluated the bioavailability of Se extracted from soybean isolate protein and tofu. The rats were kept in a low-Se state for a long time, and then supplemented with these two substances. The results showed that the total bioavailability of Se was about 101% in protein isolates and about 94% in tofu relative to SeMet. In this model, Se in natural Se-enriched soybeans has higher bioavailability and is an excellent dietary source of Se. Luo et al. [64] treated mice with gavage, and they demonstrated that the bioavailability of SePPs from rice wrapped in maize alcoholic protein and gum arabic was enhanced by ultrasound treatment. 

## 4. Effect of Processing on Se-Enriched Proteins/Peptides

Most Se-enriched agricultural products have to go through procedures such as milling, powdering, steaming, or frying before humans can ingest them. The processing of SePs/SePPs also involves techniques such as dissolving and drying. The content and chemical forms, bioaccessibility, bioavailability, and bioactivity of Se are affected [75,76,77].

### 4.1. Effect of Processing on the Structure of Se-Enriched Proteins/Peptides

Kápolna et al. [78] showed that roasting altered the distribution of Se in sesame seeds. Pérez et al. [79] showed that frying had a more significant effect on Se content in garlic compared with steaming and microwaving. This may be because frying causes the breakdown of Se amino acids into volatile Se compounds such as dimethyl selenide or dimethyl diselenide, and heat causes the degradation of SePs into SePPs and a form of insoluble protein aggregates. However, Lu et al. [80] showed that less Se in soybeans and grains was lost by frying than by boiling, and SeCys_2_ and SeMeCys were completely lost in boiled grains, probably because these Se species escape more easily into water. The temperature of oil and the type of species may have contributed to the different results of the two studies. Min et al. [81] compared rinsing and sterilization, and they showed that rinsing affected the total Se content, while sterilization had a more significant effect on organic Se. Different solutions affected the speciation of Se in broccoli, and pH had the biggest effect [82]. Moreover, light did not affect the stability of Se in solution, while the variety of solvent had a more significant effect. For example, acidification of the solution could increase the stability of Se to some extent [83]. Compared with other drying methods, Yang [76] suggested that freeze-drying could better maintain the stability of Se compounds, in addition to its better performance in drying Se-enriched soybeans, and it also allowed broccoli to retain more SeCys [84].

### 4.2. Effect of Processing on the Bioactivity of Se-Enriched Proteins/Peptides

Processing can cause changes in the structure of SePs/SePPs, which can affect their bioactivity. Liu and Ning [85] showed that the antioxidant capacity of SePs increased by 5.39% when the temperature was increased from 25 to 100 °C, because heat treatment disrupts the structure of SePs and exposes amino acids. Zhang et al. [86] found that microwave irradiation improved the antioxidant, antidiabetic, and tyrosinase inhibitory activities of SePPs of *Morchella esculenta.* Freeze-drying could effectively retain the SePs in broccoli while maintaining relatively good antioxidant capacity [84]. On the other hand, Fang [87] explored microwave drying to produce the highest antioxidant activity of Moringa leaves; however, on the whole, freeze-drying was a better choice for retaining better bioactivity.

### 4.3. Effect of Processing on the Digestion of Se-Enriched Proteins/Peptides

Processing can cause structural changes to SePs/SePPs, consequently changing their digestion, mainly in terms of bioaccessibility, and heat treatment can significantly affect the bioaccessibility of Se, SeMet, and SeCys_2_. Khanam and Platel [67] showed that heat treatment reduced the bioaccessibility of Se, SeMet, and SeCys_2_ in cereals, pulses, and green leafy vegetables by more than half. Khanam et al. [88] found that germination and fermentation did not affect the bioaccessibility of Se; however, after cooking, it was significantly improved. Zhou et al. [89] found that frying reduced the bioaccessibility of Se in abalone mushrooms, while boiling increased it. SeMet, the predominant organic Se in *Pleurotus eryngii*, is susceptible to processing treatments, especially high-temperature heating. Similar results were obtained in a study of cabbage, in which boiling reduced the Se(VI) content in cabbage but increased the SeMet in the intestinal extract [90]. Dong et al. [66] studied Se-enriched potatoes by boiling and frying them, and showed that both methods significantly increased the bioaccessibility of Se(IV) in tubers. The bioaccessibility of SeMeCys in boiled potatoes decreased, and SeMet and SeCys_2_ in fried potatoes were undetectable after digestion. Overall, the bioaccessibility of SePs was higher in boiled potatoes than in fried potatoes. Liu and Ning [85] reported that high pressure and microwave reduced the bioaccessibility of SePs in germinated brown rice. This might have been due to the fact that processing weakens the hydrophobic interaction of proteins, resulting in the exposure of hydrophobic groups and the aggregation and precipitation of proteins.

The above experimental results differ significantly, which may be due to the following reasons: (1) differences in plant species, (2) differences in the control of processing conditions, (3) differences in nutrient interactions, or (4) differences in the simulated in vitro gastrointestinal system.

With the development of the food industry, more new technology is being integrated for the processing of Se-enriched plants, but their effects on SePs/SePPs are seldom studied and need to be further explored.

## 5. Bioactivity of Se-Enriched Proteins/Peptides

**Figure 2:**
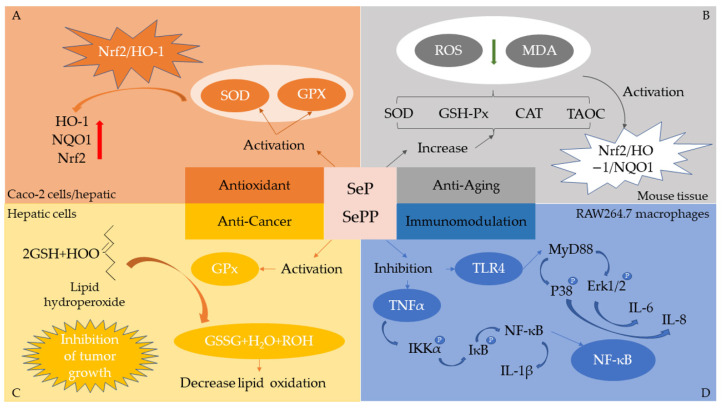
Bioactivities and related mechanisms of plant Se-enriched proteins/peptides [92,93,94,95]. (**A**) SePs has antioxidant potential via modulation of the NRF2−HO-1 signaling pathway. The soybean SePs can activate the Nrf2 pathway, leading to increases in superoxide dismutase (SOD) and gluthathione peroxidase (GPX), which are crucial for the reduction of oxidative stress. (**B**) SePPs have antiaging properties by activating the Nrf2/HO-1/NQO1 pathway. The *Cardamine violifolia* SePPs alleviate reactive oxygen species (ROS) and malonaldehyde (MDA) levels by increasing the activities of SOD, glutathione peroxidase (GSH-Px), catalase (CAT), and total antioxidant capacity (TAOC), and they activate the Nrf2/HO-1/NQO1 pathway, which probably represents the antiaging mechanism of the SePPs. (**C**) Antioxidant protection of lipids through GPx to reduce the growth of tumor. Se-enriched chickpea sprouts increased GPx activities, cholesterol, triglycerides, and low-density lipoprotein cholesterol levels to decreased significantly the tumor growth. (**D**) The regulation of NF-κB and MAPK signaling pathways by SePPs in Pb^2+^-induced RAW264.7 macrophages cells. The rice SePPs decreased the secretion levels of proinflammatory cytokines TNF-α, NF-κB, IL-1β, MyD88, IL-6, and IL-8, downregulated the expressions of IκB, IKKα, p38, and Erk1/2, and blocked the phosphorylation of these protein kinases to attenuate inflammatory response.

Se is associated with various diseases such as cardiopathy, hepatopathy, gastropathy, and cancer. Se deficiency can cause ailments such as Creutzfeldt–Jakob disease, and excessive intake of Se can lead to poisoning, resulting in hair loss, hypothyroidism, and other symptoms. As they are primary active forms, SePs/SePPs from various sources have recently become a research hotspot, but their bioactivity and mechanisms are being explored and have yet to be fully elucidated (Figure 2). In plants, Se exists in the active sites of various proteins and exerts its bioactive effects after it has been transformed into selenomethionine [91]. Current studies have shown that SePs/SePPs have diverse physiological activities such as antioxidant, anticancer, neuroprotective, immunomodulatory, anti-inflammatory, and anti-fatigue (Table 4).

### 5.1. Antioxidant

Oxidative damage is widespread in plants and animals. Se is a component of antioxidant enzymes and glutathione peroxidase [103], which can directly or indirectly reduce the levels of reactive oxygen metabolites (hydroperoxide) and protect cells from oxidative damage, while SePs/SePPs can act as antioxidants with strong nucleophilic and electron transfer capacity [4]. SePs/SePPs can scavenge double-electron oxidants, increase the rate of oxide repair, bind with metal ions, and serve as an active catalytic residue in a variety of protective enzymes to realize their antioxidant activity in various forms [104].

Se-enriched soybean isolate suppresses oxidative stress by regulating the NRF2–HO-1 signaling pathway and upregulating the expression of downstream antioxidant enzymes (GPx and SOD). Zhao et al. [92] showed that soybean SePs increased the activity of key antioxidant enzymes superoxide dismutase (SOD) and GPx in Caco-2 cells and increased HO-1, NQO1, and NRF2 levels. In animal experiments, GPx and SOD activity in mouse liver also improved with increasing soybean SePs. Soybean SePs can reduce oxidation by activating the NRF2 pathway, leading to increased SOD and GPx levels. Zhao et al. [70], using a D-galactose aging mouse model, found that soybean SePPs elevated hepatic SOD and GPx-1, while aspartate aminotransferase (AST), alanine aminotransferase (ALT), and NF-κB were significantly reduced. This indicates that soybean SePPs can inhibit oxidative stress in brain tissue by modulating the MAPK/NF-κB pathway.

### 5.2. Anticancer

The risk of cancer in Se-deficient populations is higher than in the general population, and the antitumor effects of Se are thought to be mediated through Se-binding protein 1 (SELENBP1). Its level is associated with the degree of tumor differentiation and the presence of lymph node metastasis, and it is a potential biomarker for predicting gastric cancer [105]. SELENBP1 can bind to Delta-like ligand 4 (DLL4) and antagonize the DLL4/Notch1 signaling pathway, inhibiting rectal cancer angiogenesis [106]. SELENBP1 expression is regulated by estrogen, and the inhibitory effect of Se treatment on cell proliferation is dependent on high levels of SELENBP1 expression. SELENBP1 was reduced in breast cancer tissues compared to normal controls; hence, the expression level of SELENBP1 can be used as an essential indicator to predict the efficacy of Se supplementation and survival in breast cancer patients [107].

Se can prevent cancer by reacting with tumor-promoting lipid peroxidation [108]. Accelerated oxidation motion of lipids in cancer cells leads to increased lipid peroxidation and oxidative stress. The inhibition of lipid oxidation can reduce tumor growth by blocking the supply of lipids necessary for cell proliferation [109]. Daniela et al. [94] showed that the lipid levels in an experimental group of mice were lower than the basal lipid levels in other mice, and high dietary Se intake elevated triglycerides (TRIGL), low-density lipoprotein-C (LDL-C), and cholesterol (CHOL) to near basal levels, which is an indicator of reduced lipid consumption by cancer cells.

### 5.3. Neuroprotective

Alzheimer’s disease (AD), commonly known as dementia, is a neurodegenerative disease with clinical symptoms of gradual loss of memory and life skills, and cognitive decline [110]. Its related mechanisms are still under study, with popular hypotheses including β-amyloid polypeptide cascade, Tau protein, oxidative stress, inflammatory response, neurovascular, and metabolic causes [111,112,113]. Many selenoproteins are abundantly expressed in brain tissue and have a subtle relationship with AD [114]. For example, selenoprotein M is stably overexpressed in HT22 hippocampal cells and C8-D1a cerebellar cells, and it prevents hydrogen peroxide-induced oxidative damage, while the reduction in or removal of selenoprotein M leads to decreased cell survival and increased reactive oxygen species (ROS). Thus, selenoprotein M is an antioxidant protein with neuroprotective effects [115]. Wu et al. [99] investigated the neuroprotective effects of rice SePPs on Pb^2+^-induced oxidative stress in mouse hippocampal HT22 cells. They found that SePPs increased SOD and GSH-Px activity, improved cell survival, and reduced apoptosis, indicating that SePPs inhibited Pb^2+^-induced oxidative damage. Yu et al. [93] showed that *Cardamine violifolia* SePPs could improve metabolic impairment, memory disorders, and hippocampal neuronal damage induced by D-galactose. This may be because poppy SePPs alleviate ROS and MDA levels by increasing SOD, GSH-Px, CAT, and TAOC activity and activating the Nrf2/HO-1/NQO1 pathway.

Parkinson’s disease (PD) is also a neurodegenerative condition, mainly characterized by severe motor dysfunction [116]. Its pathogenesis is likewise unexplored, with hypotheses including oxidative stress, proteasome dysfunction, and mitochondrial damage [117]. ROS are considered key regulators of PD, and, when the antioxidant capacity of cells under oxidative stress (OXS) is reduced, free radicals can severely damage or cause the death of dopamine-producing cells [118]. Chang et al. [119] showed that N-γ-(L-glutamyl)-L-selenomethionine (Glu-SeMet) improved intracellular ROS levels and was neuroprotective in a nematode PD model. Its anti-PD effect was associated with SKN-1/Nrf2 and TRXR-1.

### 5.4. Immunomodulation

Se performs well in immunomodulation [120]. Many selenoproteins can be expressed in immune cells and are essential for human immune regulation. A decrease in selenoprotein levels can affect immune cell development and immune regulation [121]. Fang et al. [122] isolated SePPs from rice, which increased the phagocytosis of macrophages and was positively correlated with selenoprotein concentration. Wu et al. [95] found that rice SePPs could effectively downregulate the expression of IκB, IKKα, p38, and Erk1/2, and successfully blocked the phosphorylation of these protein kinases, alleviating the apoptosis of RAW264.7 macrophages induced by Pb^2+^. Macrophage proliferation and phagocytosis were enhanced, immunoglobulin IgA and IgG levels were increased, and the humoral immunity of mice was significantly improved. Zhang et al. [20] found that soybean SePPs significantly increased total protein, albumin, leukocyte, white blood cell, and immunoglobulin (Ig) M, IgG, and IgA levels in blood and attenuated the atrophy of immune organs and weight loss in mice, indicating that soybean SePPs were more effective than soybean SePs in immune function.

### 5.5. Other Bioactivities

SePs/SePPs are considered to have certain antiaging functions due to their antioxidant activity [9], while numerous studies have shown that they also have various functions such as anti-inflammatory, anti-fatigue, hypotensive, and antifibrotic activity. Brown rice SePPs can act via the NF-κB/MAPK signaling pathway on LPS-induced inflammation in RAW264.7 macrophages. SePPs with a size of 1.0–3.5 kDa exhibited optimal anti-inflammatory activity by inhibiting the production of nitric oxide, prostaglandin E2, and proinflammatory cytokines [100]. Cardamom SePPs prolonged weight-bearing swimming of mice, while reducing blood lactate and urea nitrogen levels and elevating liver glycogen levels, with specific anti-fatigue effects [19]. There are many associations between ROS and hypertension, and selenoprotein has antioxidant effects that can enhance the body’s defense against ROS, thus reducing the damage to organs caused by hypertension [123]. Green tea SePs demonstrated a preventive effect and regulatory mechanism in hypertension induced by a high-salt diet in rats while protecting tissues such as the heart, liver, and kidney from damage [97]. Serum selenoprotein P can also be a diagnostic indicator of pulmonary hypertension [124]. Soybean SePPs can attenuate liver fibrosis [125], and liver fibrosis in mice induced by tetrachloromethane (CCl_4_) was found to be significantly reduced by inhibiting α-smooth muscle actin synthesis, promoting GSH-Px synthesis, and increasing mRNA expression of matrix metalloproteinase 9 in liver tissue [98].

## 6. Exploitation and Utilization of Se-Enriched Proteins/Peptides

Se resources are rare [126], but Se deficiency affects the health of hundreds of millions of people. The recommended nutrient intake (RNI) of Se is 70 μg/day for men and 60 μg/day for women; for lactating women, the RNI is 75 μg/day [127]. Some research has shown that Se intake is suboptimal in many areas, including Europe and the Middle East [128], Malawi [129], and China [130]. The dose and form of Se will affect its safety [131]. For most individuals, a good way to increase the intake of Se is through Se-enriched foods, functional foods, or supplements [132]. As species with abundant resources, plants enriched with Se, especially SePs/SePPs, are ideal sources for Se supplementation. Researchers have paid more attention to their development and obtained some results on SePs/SePPs; related products and industries are also forming.

The encapsulation of rice SePPs with zein and gum arabic, combined with ultrasound treatment, resulted in an encapsulation efficiency of 59.9% and a cumulative release rate of 80.69% from in vitro digestion, improving its bioavailability [64]. Similarly, xanthan gum and lysozyme were also considered to be good delivery systems for the encapsulation of rice SePPs, with encapsulation efficiency of 34.35% and 41%, respectively. Furthermore, the stability and antioxidant activity of rice SePPs improved because of the encapsulation [69]. More stable Se nanoparticles can be prepared in an ascorbic acid–sodium selenite reduction system using a peanut meal SePP mixture [133].

Se-enriched fish feed improved the growth rate, feed efficiency, protein retention, and muscle protein content of rainbow trout, and it had a catalytic effect on muscle protein synthesis [134]. Adding Se to cow feed can lead to a significant increase in the Se content of milk [135]. Adding Se to a moderately reduced energy and protein diet improved antioxidant status and meat quality without affecting growth performance in pigs, resulting in cost savings while improving the quality of the meat [136]. Adding a moderate amount of Se to feed is beneficial for aquatic, livestock, and poultry products, but the main types of Se used at present are yeast or inorganic Se. It may be possible to use Se-enriched plants as feed or develop feed on the basis of SePs/SePPs.

## 7. Summary and Prospect

As vital forms of Se, SePs/SePPs are highly efficient, have low toxicity, and are rich in bioactivity, and food is an essential way for humans to consume nutrients. To date, most studies have focused on preparing and identifying SePs/SePPs. Only a few analyzed their bioaccessibility and bioavailability, whereas they did not provide an in-depth analysis of their mechanisms. To better utilize and exploit the nutritional functions of SePs/SePPs, future research should focus on four aspects: first, exploring ideal sources of SePs/SePPs and developing suitable isolation and preparation techniques; second, investigating the effects of the typical processing steps on their structure and function; third, researching the mechanisms affecting their bioaccessibility and bioavailability; fourth, combining SePs/SePPs in production to develop products with better market prospects.

## Figures and Tables

**Table 1:** Liquid-phase extraction method of plant Se-enriched proteins.

Extraction Methods	Specific Processes	Application Examples	Advantages/ Disadvantages
Water extraction	The sample is crushed and added to distilled water at a certain ratio, extracted at a certain extraction temperature with stirring for a certain time, and centrifuged; the clear liquid is collected. Ammonium sulfate is added to saturate the precipitation degree and separate the precipitation fraction before dialysis, before drying to obtain water-soluble protein samples.	Cordyceps militaris [7] *Lentinus edodes* [8] Rice [9] Brazil nut [10] Garlic [11]	Advantages: better protection of biological activity of SePs; no pollution; simple operation Disadvantages: low extraction rate; time-consuming
Alkali extraction	The sample is crushed and extracted with a certain concentration of alkali at a certain temperature with stirring, the supernatant is centrifuged, and the pH is adjusted to the isoelectric point, before centrifuging and drying to obtain the protein samples.	Cordyceps militaris [7] *Lentinus edodes* [8] Mushroom [12] Rice [9] Peanut [13] Tea [14] Buckwheat [15] Quinoa [15]	Advantages: high extraction rate; suitable for the extraction of most plant SePs Disadvantages: high alkali concentration can cause protein to produce lysine, which changes its physiological function; time-consuming
Salt extraction	The sample is crushed and extracted with a certain concentration of NaCl solution at a certain temperature with stirring, the supernatant is centrifuged, and a certain amount of sulfuric acid is added to saturate the precipitation, before centrifuging and drying after dialysis to obtain the protein samples.	Cordyceps militaris [7] *Lentinus edodes* [8] Rice [9] *Ganoderma lucidum* [16]	Advantages: suitable for salt-soluble protein extraction; often used for further processing of residue after water-soluble protein extraction Disadvantages: low extraction rate; more suitable for animal-derived SeP extraction
Alcohol extraction	The sample is crushed and added to 75% ethanol at a certain ratio, extracted at room temperature with stirring, and centrifuged at 4 °C. The supernatant is added to a certain amount of distilled water and left to rest overnight, centrifuged at 4 °C to obtain the precipitate, and dried to obtain the protein samples.	Cordyceps militaris [7] Rice [9] *Ganoderma lucidum* [16]	Advantages: suitable for alcohol-soluble protein extraction; good effect with alkali method Disadvantages: low extraction rate using the method alone; easy loss of protein; organic solvents lead to pollution
Buffer solution Extraction	The sample is crushed and extracted with a certain concentration of liquid buffer (PBS, Tris-HCl, etc.) at a certain temperature with stirring, the supernatant is centrifuged, the pH is adjusted to precipitate the protein, and the precipitate is dried to obtain the protein samples.	Mushroom [12] Buckwheat [15] Quinoa [15] *Flammulina velutipe* [17]	Advantages: less damage to protein; suitable for soluble protein extraction Disadvantages: need to use organic solvent or other reagents for protein precipitation treatment

**Table 2:** Structural formulas of common seleno-amino acids.

Name	Structure
Selenomethionine, SeMet	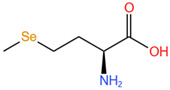
Selenocystine, SeCys_2_	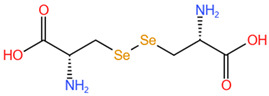
Selenocysteine, SeCys	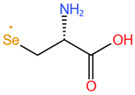
Se-methylselenocysteine, SeMeSeCys	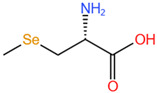

**Table 3:** In vitro and in vivo evaluation of selenium in plants.

Source	Evaluation Methods	Evaluation Object	Results	Reference
Mushroom	Gastrointestinal digestion	Bioaccessibility	75% of the Se in the mushroom was potentially bioaccessible, and SeMet was the main form	[63]
Rice	Gastrointestinal digestion	Bioaccessibility	80.69% of the rice SePPs were potentially bioaccessible	[64]
Adzuki bean sprouts	Gastrointestinal digestion	Bioaccessibility	100% of the Se in the Adzuki bean sprouts was potentially bioaccessible	[65]
Potato	Gastrointestinal digestion	Bioaccessibility	The bioaccessibility of total Se, Se(IV), SeMet, SeCys_2_, and SeMeCys was 18.3%, 32.3%, 7.0%, 23.6%, and 27.6%, respectively	[66]
Cereals; pulses; green leafy vegetables	Gastrointestinal digestion	Bioaccessibility	The bioaccessibility of Se in the cereals, pulses, and green leafy vegetables was 10–24%, 12–29%, 10–30%, respectively	[67]
Wheat	Gastrointestinal digestion; Caco-2 cells	Bioaccessibility Bioavailability	62.6–82.3% of the Se in the wheat was potentially bioaccessible, and only 19.6% was bioavailable	[68]
Rice	Gastrointestinal digestion; Caco-2 cells		91.5% and 38.95% of the two rice SePPs (TSeMMM and SeMDPGQQ) were potentially bioaccessible, and the apparent permeability coefficient of the two was 0.58 × 10^−6^ cm/s and 1.060.58 × 10^−6^ cm/s	[69]
Soybean	Caco-2 cells	Bioavailability	The transport rate of Se was 9.467% ± 0.97%	[70]
Soybean	Caco-2 cells	Bioavailability	The transport rate of soybean SePPs was 8.63% ± 1.41%	[71]
Soy protein isolate; tofu	Se-deficient rats	Bioavailability	The bioavailability of soy protein isolate and tofu was 101% and 94%, relative to SeMet	[72]

**Table 4:** The Biological activities of plant Se-enriched proteins/peptides.

Source	Bioactivities	Identification Approach	Se Status	Sequence	Reference
Broccoli sprout	Anticancer	HPLC/ICP-MS	SeMeSeCys	Not studied	[96]
*Cardamine* *violifolia*	Anti-fatigue Antioxidant	LC–MS/MS	SeCys SeMet MeSeCys	Tyr–Leu–Pro–Gly–SeMet–Val; Phe–SeCys–Leu–Val–Glu–Ser–Thr; Val–His–Thr–SeCys–Pro–Ile–SeCys–Thr–Ser; Leu–Leu–Thr–MeSeCys–Pro–Ala; Ser–Val–Ile–Ala–Thr–Ile–SeMet–Val–Pro; Ser–SeCys–SeCys–Ser–Pro–Thr–Pro; Lys–Lys–SeCys–Ser–Leu; Cys–Pro–Gln–Ser–MeSeCys–Lys; Asn–SeCys–Val–Ala–Ser–Pro–Leu; Asn–Leu–Ile–Val–Asn–SeMet–Lys–Asn	[19]
Green tea	Antihypertensive	Not studied	Not studied	Not studied	[97]
Soybean	Hepatoprotective	HPLC–ESI-MS/MS	SeCys SeMet	SeMet–Val–Val–SeCys; Ser–SeCys–Arg–Asp–Cys–Val; Phe–Ile/Leu–Phe–SeCys–Phe; SeCys–Ile/Leu–Ser–SeCys	[98]
Rice	Antiaging	Not studied	Not Studied	Not Studied	[9]
Rice	Immunomodulatory	RP-UPLC triple-TOF MS/MS	SeMet MeSeCys	SeMet–Pro–Ser; Met–MeSeCys–Glu; SeMet–MeSeCys–Glu	[95]
Rice	Immunomodulatory	RP-UPLC triple-TOF MS/MS	SeMet	SeMet–Aps–Pro–Gly–Gln–Gln; Thr–SeMet–Met–Met	[18]
Rice	Neuroprotective	RP-UPLC triple-TOF MS/MS	SeMet	SeMet–Aps–Pro–Gly–Gln–Gln; Thr–SeMet–Met–Met	[99]
*Cardamine* *violifolia*	Neuroprotective	Not studied	Not studied	Not studied	[93]
Brown rice	Anti-inflammatory	Triple-TOF LC-MS/MS	Not studied	Ala–Leu–Leu–Leu–Gln–Ala–Val–Gln–Ser–Gln–Tyr–Glu–Glu–Lys	[100]
Soybean	Antioxidant	Not studied	Not studied	Not studied	[92]
Walnut	Antioxidant	LC–HG-AFS	MeSeCys SeMet SeCys_2_	Not studied	[101]
*Ganoderma lucidum* mushroom	Antioxidant	Not studied	Not studied	Asp–Ile–Asn–Gly–Gly–Gly–Ala–Thr–Leu–Pro–Gln–Lys–Leu–Tyr–Leu–Thr–Pro–Asp–Val–Leu	[102]

## Data Availability

Not applicable.
